# Sodium thiosulfate during cisplatin-based hyperthermic intraperitoneal chemotherapy is associated with transient hypernatraemia without clinical sequelae

**DOI:** 10.1515/pp-2022-0107

**Published:** 2022-05-02

**Authors:** Anais Alonso, Winston Liauw, Helen Kennedy, Nayef A. Alzahrani, David L. Morris

**Affiliations:** Liver and Peritonectomy Unit, St George Hospital, Kogarah, Australia; St George and Sutherland Clinical School, University of New South Wales, Kogarah, Australia; College of Medicine, Al Imam Mohammad Ibn Saud Islamic University, Riyadh, Saudi Arabia

**Keywords:** acute kidney injury, cisplatin, cytoreductive surgery, hypernatraemia, hyperthermic intraperitoneal chemotherapy, sodium thiosulfate

## Abstract

**Objectives:**

Cisplatin is commonly used during intraperitoneal chemotherapy however has well-established nephrotoxic side-effects. Sodium thiosulfate is often added to cisplatin-based hyperthermic intraperitoneal chemotherapy (HIPEC) protocols to mitigate this, however evidence regarding risk of hypernatraemia is scarce as of yet.

**Methods:**

We retrospectively identified patients undergoing cytoreductive surgery (CRS) for peritoneal surface malignancies of any origin at a single high-volume unit between April 2018 and December 2020. Patients were included if they received cisplatin-based HIPEC with intravenous sodium thiosulfate. Blood tests were collected pre-surgery and then daily during admission. Hypernatraemia was defined as serum sodium >145 mmol/L. Renal impairment was defined using the RIFLE criteria.

**Results:**

Eleven CRSs met inclusion criteria, the majority of which were indicated for ovarian cancer (72.7%). One (9.1%) patient with mesothelioma received mitomycin C as an additional chemotherapy agent. The incidence of hypernatraemia was 100% but all cases were transient, with no clinical sequelae observed. The rate of AKI was 36.4%, with three (27.3%) patients classified as risk and one (9.1%) instance of failure. No long-term renal impairment was observed.

**Conclusions:**

Despite biochemical evidence of mild hypernatraemia but with the absence of clinical sequelae, sodium thiosulfate appears to be safe when used in adjunct to cisplatin-based HIPEC during CRS. These findings should be evaluated with further comparative studies. When describing renal impairment, it is important that standardisation in reporting occurs, with the RIFLE and Acute Kidney Injury Network criteria now the preferred consensus definitions.

## Introduction

While cytoreductive surgery (CRS) allows for removal of macroscopic deposits of peritoneal surface malignancies (PSM), perioperative intraperitoneal chemotherapy (PIC) is necessary to eliminate microscopic residual tumour, minimising systemic side effects [1] and improving survival compared to systemic intravenous chemotherapy regimes [2, 3].

Cisplatin is one of the most common intraperitoneal chemotherapy agents, administered to patients with mesothelioma, ovarian cancer and sarcomas. However, its nephrotoxic effects are well-established and are a major dose-limiting factor for both intravenous and intraperitoneal cisplatin-based chemotherapy [4]. Renal impairment of varying severity has been reported in 3.7–48.0% of patients receiving cisplatin-based PIC [5, 6]. The mechanism of nephrotoxicity is not entirely understood, but is theorised to occur due to induction of apoptosis and necrosis of epithelial cells in the pars recta of the proximal tubule at low and high concentrations of cisplatin respectively [7, 8]. Additionally, cisplatin-induced injury of the renal vasculature may result in further ischaemic damage [9]. These nephrotoxic effects begin on day three post-cisplatin administration and peak on day five [8], and are cumulative and dose-dependent [4], though not always reversible [10]. As such, a nephroprotective protocol is crucial to maximise chemotherapeutic potential but mitigate risk to the patient.

The nephroprotective properties of sodium thiosulfate are well-documented, which has been administered concurrently with intravenous systemic cisplatin-based chemotherapy [11, 12]. The mechanism explaining this is unclear but has been proposed to result from systemic induction of inactive cisplatin metabolite formation, reducing the concentration of unchanged cisplatin filtered by the kidney [13]. Evidence supporting its use in PIC is more limited, with a handful of small studies comparing rates of renal impairment following cisplatin-based HIPEC with or without sodium thiosulfate [14], [15], [16]. Following publication of the OVIHIPEC trial [17], intravenous sodium thiosulfate has been included in our cisplatin-based HIPEC protocol since April 2018.

One concern regarding intravenous sodium thiosulfate is the potential to cause hypernatraemia. At sufficiently high levels, hypernatraemia is associated with significant morbidity, including seizures, coma, intracranial haemorrhage and demyelinating lesions, as well as mortality in extreme cases [18]. A potential mechanism for hypernatraemia secondary to sodium thiosulfate has not been proposed in the literature but is likely due to direct sodium overload owing to the solution’s hypertonicity. Two small studies in which intravenous carboplatin and sodium thiosulfate were administered reported transient hypernatraemia, even at small doses [19, 20]. Evidence regarding the risk of hypernatraemia following sodium thiosulfate is scarce in cases of cisplatin-based chemotherapy, including PIC, where patients are at increased risk of fluid loss and electrolyte derangements due to several factors, including longer operating times and a greater area of exposed abdominal cavity [21]. One retrospective study investigating the reduction in acute kidney injuries (AKIs) following sodium thiosulfate during CRS with cisplatin-based HIPEC reported an increase in serum sodium without clinical sequelae [22].

The aim of this study is to report preliminary data pertaining to sodium thiosulfate during CRS with cisplatin-based HIPEC. The primary outcome is the incidence of hypernatraemia, with the rate of AKI measured as a secondary endpoint. Other associated electrolytes, including magnesium, phosphate and calcium, are also reported.

## Materials and methods

### Patient selection

The research related to human use has complied with all the relevant national regulations, institutional policies, and in accordance with the tenets of the Helsinki Declaration, and has been approved by St George Hospital’s Human Research Ethics Committee (approval number 18/078). Selection of candidates for CRS performed at St George Hospital, Sydney, Australia, including pre-operative work-up and management, is described elsewhere by our unit [23]. Data pertaining to CRS performed at our institution are collected in a prospectively maintained database. Patients undergoing CRS between April 2018 and December 2020 for PSM of any origin with peritoneal cancer index (PCI) ≥1 and who received cisplatin-based HIPEC were identified from our database. Of these patients, those who received intravenous sodium thiosulfate at the time of CRS according to the operation report were included in the study. For the primary outcome, a control group is included, consisting of the equivalent number of most recent patients to undergo CRS for PSM of any origin with PCI ≥1 and receive cisplatin-based HIPEC prior to the commencement of our sodium thiosulfate protocol.

### Intraoperative care

CRS was performed using Sugarbaker’s technique [24], with HIPEC subsequently administered in a standardised fashion. The cisplatin-based HIPEC protocol utilised by our unit is described elsewhere [25]. In short, cisplatin (100 mg/m^2^ in 1,000 mL 0.9% sodium chloride) was administered intra-abdominally at 41.5°C for 90 min via the open Coliseum technique. Patients with peritoneal mesothelioma also received mitomycin C (12.5 g/m^2^) in addition to cisplatin. Cisplatin was dose-reduced by 25% in patients with pre-existing renal impairment, or a history of prior HIPEC or systemic chemotherapy with more than one agent.

A nephroprotective sodium thiosulfate protocol was introduced at our institution in April 2018 following publication of the OVIHIPEC trial [17]. Patients undergoing cisplatin-based HIPEC were administered sodium thiosulfate (9 g/m^2^ in 200 mL 0.9% sodium chloride) via intravenous bolus at the commencement of HIPEC, followed immediately by a second intravenous infusion (12 g/m^2^ in 1,000 mL 0.9% sodium chloride) over 6 h. Patients did not receive further sodium thiosulfate doses either intra- or post-operatively.

Patients were monitored intraoperatively, with intravenous fluid management titrated to ensure that urine output remained greater than 0.5 mL/kg/h. Colloid or blood were administered as clinically indicated.

### Post-operative care

Our institution’s post-operative protocol is described elsewhere [25]. In short, all patients were admitted to the intensive care unit (ICU) immediately post-CRS, where intravenous fluid management was similarly titrated to achieve a urine output of greater than 0.5 mL/kg/h. Patients were transferred to the ward when clinically indicated, where strict fluid balance monitoring continued. Daily blood tests, including renal function and electrolytes, were collected via venepuncture at approximately 6 am. Repeat blood tests were collected from patients requiring further monitoring. Hypernatraemia and renal impairment were treated with intravenous fluids as clinically indicated, including 0.9% sodium chloride, Harmann’s solution and 5% dextrose, with additional electrolyte replacement if depleted.

### Statistical analyses

Descriptive statistics were reported using the mean and standard deviation (SD), or frequency, as appropriate. Owing to the small sample size and subsequent risk of type two statistical error, comparative statistical analyses were not undertaken.

Baseline pathology results were collected on the day prior to surgery. All pathology results were derived from laboratory analyses and not point of care testing. If multiple blood tests were collected in a single day, the first test was used for statistical analyses. Normal electrolyte concentrations ranges are defined in Table 1. Calcium was corrected for albumin using the formula: corrected calcium (mmol/L) = total calcium (mmol/L) + 0.02 (40 – albumin [g/L]). AKI was defined according to the RIFLE criteria (Table 2) [26], considering only the creatinine and glomerular filtration rate (GFR) criteria, since data pertaining to urine output were unavailable. Estimated GFR (eGFR) was calculated using the Chronic Kidney Disease Epidemiology Collaboration (CKD-EPI) equation [27]. Perioperative morbidity was categorised using the Clavien-Dindo classification of surgical complications [28]. In-hospital mortality was defined as death during the same hospital admission as that for CRS. Four patients in the intervention group and seven in the control group were discharged prior to one month post-CRS hence were excluded from month 1 analyses in Tables 4 and 5.

**Table 1:** Normal electrolyte ranges.

Electrolyte	Normal range, mmol/L
Sodium	135–145
Magnesium	0.70–1.10
Phosphate	0.75–1.50
Corrected calcium	2.10–2.60

**Table 2:** RIFLE criteria.

Category	Creatinine/GFR criteria	UO criteria
Risk	Increased serum creatinine ×1.5 or GFR decrease >25%	UO <0.5 mL/kg/h for 6 h
Injury	Increased serum creatinine ×2 or GFR decrease >50%	UO <0.5 mL/kg/h for 12 h
Failure	Increased serum creatinine ×3 or serum creatinine ≥4 mg/dL or GFR decrease >75%	UO <0.3 mL/kg/h for 24 h or anuria for 12 h
Loss	Persistent acute renal failure or complete loss of renal function for >4 weeks	
ESKD	ESKD for >3 months	

ESKD, end-stage kidney disease; GFR, glomerular filtration rate; UO, urine output.

## Results

Between April 2018 and December 2020, 11 CRSs performed by our unit met inclusion criteria. Complete patient and peri-operative characteristics are described in Table 3. One patient with mesothelioma received mitomycin C in addition to cisplatin-based HIPEC. None of the 11 patients received systemic intravenous or early postoperative intraperitoneal chemotherapy during their surgical admission. There were no events of in-hospital mortality.

**Table 3:** Patient demographics and clinical characteristics (n=11).

Age (years), mean ± SD	60.2 ± 12.1
Sex, n (%)	
Male	2 (18.2)
Female	9 (81.8)
BMI (kg/m^2^), mean ± SD	28.6 ± 7.1
Diagnosis, n (%)	
Endometrial carcinoma	1 (9.1)
Mesothelioma	1 (9.1)
Ovarian cancer	8 (72.7)
Sarcoma	1 (9.1)
Previous CRS, n (%)	2 (18.2)
Operating time (hours), mean ± SD	9.75 ± 2.0
PCI, mean ± SD	25.7 ± 12.4
CC score, n (%)	
0	7 (63.6)
1	1 (9.1)
2	3 (27.3)
Additional HIPEC agent, n (%)	1 (9.1%)
Length of ICU admission (days), mean ± SD	9.4 ± 13.0
LoS (days), mean ± SD	34.0 ± 22.6
Grade III-IV morbidity, n (%)	7 (63.6)

BMI, body mass index; CC, completeness of cytoreduction; CRS, cytoreductive surgery; HIPEC, hyperthermic intraperitoneal chemotherapy; ICU, intensive care unit; LoS, length of stay; PCI, peritoneal cancer index; SD, standard deviation.

### Hypernatraemia

Pre-operatively, serum sodium values were within normal limits for all patients. The incidence of post-operative hypernatraemia was 100% (n=11), all cases of which were detected immediately post-CRS. Sodium values were highest immediately post-CRS (mean 149.8 mmol/L; SD 2.8), while the mean of each patient’s peak sodium concentration was 150.4 mmol/L (SD 2.7). Hypernatraemia resolved by post-operative days 1–8 (median day three).

In the control group, one patient was hypernatraemic pre-operatively (sodium concentration 146 mmol/L), and remained hypernatraemic immediately post-operatively (sodium concentration 147 mmol/L). This patient was eunatraemic by post-operative day one (sodium concentration 145 mmol/L) but recorded a serum sodium concentration of 146 mmol/L on post-operative day two, which again resolved by post-operative day three. Of the remaining patients, eight developed hypernatraemia, however the mean of each patient’s highest sodium concentration was lower than in patients receiving sodium thiosulfate (145.5 mmol/L; SD 2.4). Hypernatraemia onset was also delayed compared to the intervention group, with day two being the only day on which the mean serum sodium concentration exceeded 145 mmol/L (mean 145.5 mmol/L; SD 3.6).

In both groups, no clinical sequalae of hypernatraemia or its management were observed. No further diagnoses of hypernatraemia were made during surgical admission. Further details are provided in Table 4.

**Table 4:** Sodium results.

	Serum sodium (mmol/L), mean ± SD	
	Sodium thiosulfate (n=11)	Control (n=11)
Baseline	140.0 ± 3.0	141.0 ± 2.7
Post-surgery	149.8 ± 2.8	144.2 ± 2.4
Day 1	148.6 ± 2.8	144.9 ± 2.5
Day 2	145.5 ± 4.4	145.5 ± 3.6
Day 3	144.1 ± 4.9	143.6 ± 4.1
Day 7	139.7 ± 3.7	138.1 ± 4.7
Month 1	136.7 ± 3.4	136.5 ± 1.7

SD, standard deviation.

### Acute kidney injury

Pre-operatively, all patients exhibited normal renal function, with no pre-existing diagnoses of acute or chronic kidney disease. Post-operatively, AKI was diagnosed in 4/11 (36.4%) patients. According to the RIFLE criteria, three (27.3%) patients were classified as Risk, identified on post-operative days zero (immediately post-CRS), one and six. Renal impairment resolved on days five, three and eight respectively. One (9.1%) patient was classified as failure, identified on post-operative day two. His renal function normalised on post-operative day four. Of the patients still admitted to hospital one month post-CRS, none had ongoing renal impairment. Of the patients who developed an AKI, hypernatraemia was still persistent at the time of diagnosis of AKI in 3/4 (75%) cases (Table 5).

**Table 5:** Further laboratory results (n=11).

	Baseline	Post-surgery	Day 1	Day 2	Day 3	Day 7	Month 1
eGFR (mL/min/1.73 m^2^), mean ± SD	88.9 ± 11.6	87.7 ± 12.9	83.2 ± 20.2	79.4 ± 23.4	90.6 ± 19.5	98.4 ± 14.0	95.3 ± 9.8
Serum magnesium (mmol/L), mean ± SD	0.8 ± 0.1	0.7 ± 0.1	1.2 ± 0.4	1.1 ± 0.2	0.9 ± 0.2	0.7 ± 0.1	0.8 ± 0.1
Serum phosphate (mmol/L), mean ± SD	1.2 ± 0.2	1.3 ± 0.4	1.5 ± 0.6	1.0 ± 0.5	0.8 ± 0.3	1.0 ± 0.2	1.1 ± 0.1
Corrected calcium (mmol/L), mean ± SD	2.4 ± 0.1	2.2 ± 0.2	2.0 ± 0.1	2.1 ± 0.1	2.2 ± 0.1	2.3 ± 0.1	2.4 ± 0.1

eGFR, estimated glomerular filtration rate; SD, standard deviation.

## Discussion

Cisplatin-induced nephrotoxicity involves complex mechanisms of cellular death, resulting in proximal renal tubule and vascular damage [7], [8], [9], and is a major dose-limiting factor for both intravenous and intraperitoneal cisplatin chemotherapy [4]. The nephroprotective properties of sodium thiosulfate are well-documented, but adverse outcomes, including hypernatraemia, are not extensively described in the literature.

Our preliminary data suggest that transient hypernatraemia is universal in patients receiving sodium thiosulfate during CRS with cisplatin-based HIPEC, with an incidence of 100%. These are novel data, with no studies exploring dysnatraemias following CRS with cisplatin-based HIPEC, regardless of whether sodium thiosulfate was administered, and only one report of mild hypernatraemia in patients receiving cisplatin-based HIPEC with sodium thiosulfate in the absence of CRS, though no incidence was reported [22]. Sequelae of hypernatraemia are predominantly neurological, ranging from lethargy and weakness to seizures, coma, intracerebral and subarachnoid haemorrhages, and demyelinating lesions. Such complications are usually only observed when serum sodium rises acutely above 158 mmol/L, with the highest mortality seen above 180 mmol/L [18]. It is thus important to consider the clinical significance of hypernatraemia within our cohort; no daily mean or individual serum sodium value exceeded the lower of these values, and no sequelae of hypernatraemia were reported, supporting the safety of this sodium thiosulfate protocol despite the observed laboratory values. Of note, the incidence of hypernatraemia in the control group was still high but negligibly above the normal sodium range. This group also had a later onset of hypernatraemia, suggesting that hypernatraemia in the sodium thiosulfate group is a direct result of hypertonic saline administration but may exacerbate other causes of hypernatraemia, including hypovolaemia secondary to intraoperative losses. Any statistical differences need to be ascertained in future comparative studies, which should also investigate other data of clinical relevance, including length of stay (LoS), length of ICU admission, and morbidity and mortality. Of note, hyponatraemia is a well-known complication of systemic cisplatin, with an incidence of up to 43% [29], but no reports of this in relation to cisplatin-based HIPEC exist. In our cohort, one patient in the control group recorded a serum sodium concentration of 134 mmol/L on day three post-CRS, though was eunatraemic pre- and post-this value. No patients in the sodium thiosulfate group were hyponatraemic during the reported timeframe.

In this study, the incidence of AKI was 36.4%, as defined by the RIFLE criteria. This is comparable to rates of AKIs detected in patients undergoing CRS with cisplatin-based HIPEC [5, 6], but considerably higher than in studies in which sodium thiosulfate was also administered, all of which report an incidence of 0% [15, 17], despite identical cisplatin and sodium thiosulfate protocols. Rates of AKIs in CRS with HIPEC, regardless of chemotherapy agent, are reported to be 21.3–31.8% [30, 31]. It is possible that this discrepancy arose due to inconsistent definitions of AKI, with one study utilising the Kidney Disease Improving Global Outcomes definition [32] and the remaining two [15, 17] providing no methodology regarding their definition of AKI. Moving forward, it is important that standardisation in reporting occurs across the literature, with the RIFLE criteria, employed in our study, and Acute Kidney Injury Network (AKIN) criteria now the preferred consensus definitions. Additionally, rates of AKI in the control groups also varied markedly, with one study reporting only one (14.3%) AKI in patients receiving cisplatin (50 mg/m^2^) without sodium thiosulfate and none in patients undergoing CRS without HIPEC. In another study, the incidence of AKI was 31.4% in those receiving cisplatin without sodium thiosulfate, with chronic kidney disease (CKD) ensuing in 25.7% at three months follow-up [15]. Importantly, the former study only analysed renal function one day, three days and three months post-CRS, meaning that any renal impairment occurring between these times may have been missed, since nephrotoxic effects of cisplatin begin on day three post-administration and peak on day five [8]. However, the timing of the AKIs within our cohort may suggest an alternative aetiology to cisplatin-induced nephrotoxicity, since most of our patients who developed an AKI were diagnosed prior to this. Such causes could include haemorrhage, insensible fluid losses or nephrotoxic medications. Notably, the incidence of AKI was also substantially higher than previous reported by our group in a cohort of patients undergoing CRS with cisplatin-based HIPEC without sodium thiosulfate (15.4%), though this may be obscured by this study’s markedly smaller sample size (compared to 111 patients) [25]. It is also important to consider that our cohort exhibited a high PCI, with previous work by our group demonstrating that a higher PCI is a risk factor for developing an AKI [25]. It is important to note that whilst all AKIs documented in our study resolved during admission, the lack of long-term follow up in this study, and indeed within the literature, does not allow for consideration of chronic complications associated with cisplatin-induced nephrotoxicity, particularly CKD.

Systemic cisplatin is associated with various electrolyte disturbances, including hypomagnesaemia, with reports that magnesium supplementation is effective in reducing cisplatin-induced nephrotoxicity [33]. Only one study has described hypomagnesaemia following cisplatin-based HIPEC [5]. On the contrary, previously published data by our group demonstrated that patients who underwent CRS with cisplatin-based HIPEC but without sodium thiosulfate were more likely to have a higher serum magnesium on day three if they developed an AKI, but not above the upper limit of normal. Hypomagnesaemia in the first seven days post-CRS was not identified as a risk factor for developing an AKI [25]. Similarly, in this present study, the mean daily magnesium was above the upper limit of normal on day one only, and never below the normal range. Evidence regarding other cisplatin-associated electrolyte derangements is more limited, but includes hypocalcaemia, and hypophosphataemia in one study post-CRS with cisplatin-based HIPEC and sodium thiosulfate [34]. In our study, the mean daily corrected calcium concentration was below the lower level of normal on day one only, and mean daily phosphate concentrations were normal throughout the study period. As opposed to outpatient systemic chemotherapy regimens, to which most of the literature pertains, our surgical patients were admitted to hospital and underwent daily electrolyte monitoring, with each of these replaced before reaching a level of depletion. The nephroprotective effects of intraperitoneal chemotherapy with sodium thiosulfate as opposed to systemic cisplatin may also decrease the incidence of these electrolyte derangements, since all are common findings in patients with AKIs.

This body of work is strengthened by use of the RIFLE criteria, which has important clinical correlations with mortality, increased LoS, renal replacement therapy and recovery, and discharge from hospital to institutionalised care [35]. Contrarily, the authors acknowledge the significant limitations of this study. While the timing of hypernatraemia supports the hypothesis that it is the direct result of parenteral sodium delivery, our study cannot account for other causes, such as intravenous hypertonic sodium bicarbonate to treat metabolic acidosis, since anaesthetic data and fluid orders are not documented in the patients’ electronic medical records, or hyperglycaemia, which is a common stress response to major surgery, even in non-diabetic patients [36], and can result in dilutional hyponatraemia due to the osmotic gradient exerted by glucose [37]. This matter is further complicated by the intimate relationship between AKIs and electrolyte disturbances, including both hypo- and hypernatraemia, however the directionality of this relationship cannot be established from our retrospective data alone, noting that 75% of patients who developed an AKI had persistent hypernatraemia at the time of AKI diagnosis, with the remaining patient eunatraemic. Management instigated in response to either hypernatraemia or AKI is unavailable however, as patients were recruited from a single site, with continuity of the treating team, it can be assumed that such management is consistent between cases. Additionally, post-operative urine output was not recorded in the electronic medical records and thus is it possible that the incidence of AKI is underestimated owing to its exclusion, as this forms part of the RIFLE criteria. Finally, the small sample size rendered statistical comparison with a control group inappropriate due to the high risk of type two statistical error. These data are intended only as preliminary and to be followed up with a larger-scale study in the coming years as the number of patients undergoing this protocol at our centre expands.

In summary, we provide important preliminary data regarding the incidence of hypernatraemia following cisplatin-based HIPEC, though the statistical and clinical significance of this finding must be evaluated in future studies. The incidence of AKI was high in our cohort, and demonstrates the importance of standardisation in the reporting of outcomes, with the RIFLE and AKIN criteria preferred consensus definitions for describing renal impairment.
